# A rapid, simple questionnaire to assess gastrointestinal symptoms after oral ferrous sulphate supplementation

**DOI:** 10.1186/1471-230X-14-103

**Published:** 2014-06-04

**Authors:** Dora IA Pereira, Susana S Couto Irving, Miranda CE Lomer, Jonathan J Powell

**Affiliations:** 1 MRC Human Nutrition Research, Elsie Widdowson Laboratory, CB1 9NL Cambridge, UK; 2 Diabetes and Nutritional Sciences Division, King’s College London, London, UK

**Keywords:** Gastrointestinal symptoms, Ferrous sulphate, Iron supplementation, Side-effects, Adverse events, Oral iron

## Abstract

**Background:**

Oral iron supplementation is often associated with rapid onset of gastrointestinal side-effects. The aim of this study was to develop and trial a short, simple questionnaire to capture these early side-effects and to determine which symptoms are more discriminating.

**Methods:**

The study was a double-blind placebo-controlled randomized parallel trial with one week treatment followed by one week wash-out. Subjects were randomized into two treatment groups (n = 10/group) to receive either ferrous sulphate (200 mg capsules containing 65 mg of iron) or placebo, both to be taken at mealtimes twice daily during the treatment period. Subjects completed the questionnaires daily for 14 days. The questionnaire included gastrointestinal symptoms commonly reported to be associated with the oral intake of ferrous iron salts (i.e. nausea, vomiting, heartburn, abdominal pain, diarrhoea, and constipation).

**Results:**

Seventy five per cent of participants reporting the presence of one or more symptoms in the first week of the study were in the ferrous sulphate group. In the second week of the study (i.e. wash-out), 67% of the participants reporting one or more symptom(s) were in the ferrous sulphate group. In the first week of the study (treatment) the number of symptoms reported by participants in the ferrous sulphate group (mean ± SEM = 6.7 ± 1.7) was significantly higher than that for participants in the placebo group (1.2 ± 0.5) (*p* = 0.01). In the second week of the study (wash-out) the number of symptoms reported by participants in the ferrous sulphate group (4.6 ± 2.0) appeared higher than for participants in the placebo group (1.0 ± 0.7) although this did not reach significance (*p* = 0.12). Events for which the gastrointestinal symptom questionnaire was most discriminatory between ferrous sulphate and placebo groups were: heartburn, abdominal pain and the presence of black stools (all *p* ≤ 0.03).

**Conclusions:**

A tool for the detection of commonly-occurring side effects should not require large study numbers to be effective. With just 10 subjects per group (iron or placebo), this simple questionnaire measures gastrointestinal side-effects associated with oral iron (ferrous sulphate) supplementation, and would be appropriate for use in intervention studies or clinical trials.

**Trial registration:**

ClinicalTrials.gov Identifier: NCT02146053 (21/05/2014).

## Background

Despite many attempts to eradicate or even reduce iron deficiency anaemia (IDA), it remains the most prevalent nutritional deficiency disorder in the world affecting around 1.1 billion people of which nearly 400 million are children. The World Health Organization (WHO) reports that worldwide, at any given moment, more individuals have IDA than any other health problem [1]. Iron deficiency anaemia accounts for 2.4% of the global disease burden and it occupies the World Health Organization (WHO) top ten hit list of targeted global health problems [1-3].

One of the hurdles to eradicate this disorder has been the use of iron preparations, typically ferrous iron salts 65 mg_Fe_ 2–3 times daily [4], that risk significant gastrointestinal disturbances [5-10] and systemic infection [11-14]. Data from the National Health Service shows that at least 97.5% of the prescriptions filled for oral iron in England alone are for simple ferrous salts (sulphate, fumarate or gluconate) [15]. In the main, gastrointestinal side-effects start to present early in the treatment course [7,16,17] albeit onset of symptoms and severity must be related to the iron dosage and the posology followed (i.e. weekly or daily) [18-23]. Reasons for gastrointestinal disturbance are well described: even in subjects with IDA only about 20-30% of oral supplemented iron is absorbed, while the remainder transits through the gut lumen inducing (a) free radical mediated damage to the gut mucosa [9,24-26] and (b) undesirable changes to colonic microflora [27-29]. Gastrointestinal symptoms impact on the general wellbeing of individuals [30-37] and are known to affect compliance with oral iron therapy and, therefore, treatment efficacy [7,19,38-40].

Self-reporting questionnaires may be used to monitor gastrointestinal symptoms in disease-specific scenarios [35,41-44], although only a few have been formally tested [36], but there is currently no gastrointestinal-specific questionnaire to identify oral iron-induced symptoms in healthy subjects [45]. The current study presents a simple one-page gastrointestinal symptom questionnaire (a) to capture the early side-effects normally associated with oral iron (ferrous sulphate) supplementation and (b) to determine which symptoms are more discriminant. Since gastrointestinal side effects, following oral iron, are reportedly so common, any robust questionnaire should be valid in just a small number of subjects. With just 10 subjects per group (iron or placebo), this questionnaire is shown to be a useful instrument to measure gastrointestinal side-effects associated with oral iron supplementation, and would thus be appropriate for use in intervention studies or clinical trials.

## Methods

### Gastrointestinal symptom questionnaire

**Figure 1:**
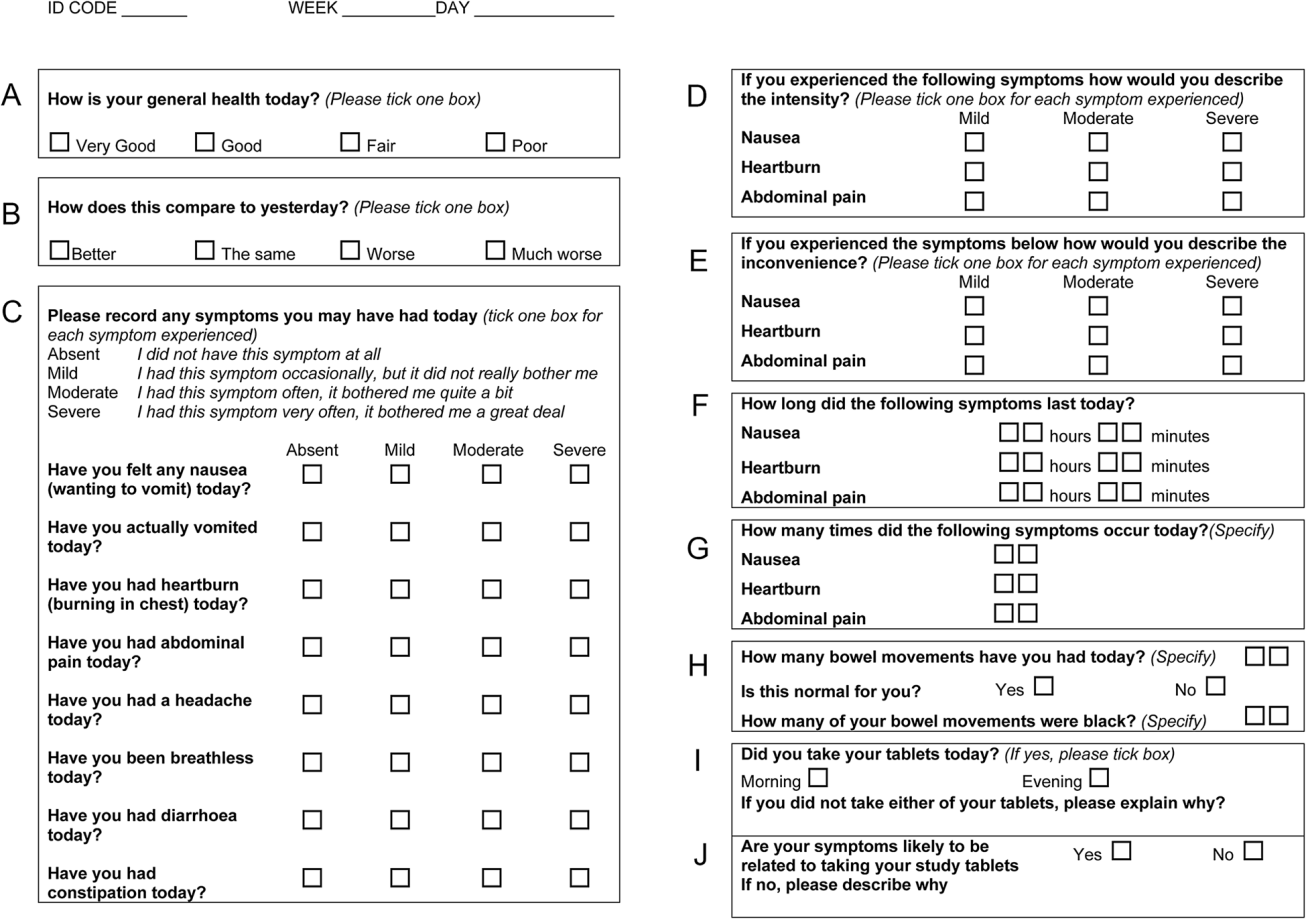
**One-page symptom questionnaire to assess gastrointestinal adverse effects after oral iron supplementation.** The letters ‘A’ to ‘J’ are not present on the actual questionnaire but are herein shown for ease of reference throughout this manuscript.

Questions for the gastrointestinal symptom questionnaire (Figure 1) were based on previously validated bowel symptom questionnaires [30,44,46,47] and included gastrointestinal symptoms commonly reported to be associated with the oral intake of ferrous iron salts: i.e. nausea, vomiting, heartburn, abdominal pain, diarrhoea, and constipation [5,19,48,49]. In addition, the questionnaire included questions on breathlessness because this is common in anaemic patients and headache that, although not generally associated with oral iron therapy, is common with some gastrointestinal complaints [50,51].

The degree of discomfort from each symptom was ranked by the study subjects in one of four categories (absent, mild, moderate or severe) as used in previous studies [44,52]. The frequency of bowel movements was registered and the questionnaire included a question on whether these were normal or abnormal. Compliance with the oral iron or placebo treatment was also assessed.

### Participants

Twenty healthy subjects, seven men and thirteen women aged 18–65 years (average age 32), were recruited via circular email. The presence of any chronic disease, pregnancy or lactation were considered as exclusion criteria. Subjects were asked to complete a baseline questionnaire including demographic questions on age, gender, education and job title.

This study was conducted according to the guidelines laid down in the Declaration of Helsinki and all procedures involving human subjects were approved by the King’s College London (KCL) Research Ethics Committee and treatment capsules were approved by the U.K. Medicines and Healthcare Products Regulatory Agency (MHRA). Written informed consent was obtained from all subjects.

### Study design

The study was a double-blind placebo-controlled randomized parallel trial with one week treatment followed by one week wash-out. Subjects were randomizsed into two treatment groups (n = 10/group) to receive either ferrous sulphate (200 mg capsules containing 65 mg of iron) or placebo, both to be taken at mealtimes twice daily for 7 days during the treatment period. All subjects were advised that they may suffer black stools, as a normal consequence of intervention, irrespective of whether they were on placebo or oral iron (although placebo had no specific stool-darkener added).Subjects were provided with two seven-day symptom questionnaires (Figure 1) to take home for self-completion, and asked to complete them on a daily basis for 14 days – the first 7 days whilst on one of the treatments (i.e. ferrous sulphate or placebo) and the following 7 days during the respective wash-out periods. The symptom diaries were coded and anonymised. Codes were broken only after the raw questionnaire data had been entered into the database.

### Data analysis

The questionnaire is broken down into different compartments (boxes ‘A’-‘J’) for ease of reference in this manuscript (Figure 1). Although all data were collected, only those from boxes C, H,I and J were analysed and are herein reported in figures, tables and/or text. All statistical analysis was performed using GraphPad Prism version 5.03 for Windows (GraphPad Software, San Diego, California, USA). Results are presented as means with either standard error of mean (SEM) or standard deviation (SD) as described. Unless otherwise stated, differences between treatment and wash-out periods and between ferrous sulphate and placebo were examined using the unpaired *t*-test (two-tailed) with Welch’s correction for non-equal variances (level of significance set to *p* < 0.05). Where, for the placebo group, there was an absence of an individual symptom (i.e. for heartburn, breathlessness or black stools) comparisons with the ferrous sulphate group were made using the paired *t*-test (two-tailed). The number of black stool movements on each day was compared using the one-way analysis of variance with the Bonferroni post-hoc test to correct for multiple comparisons.

## Results and discussion

Twenty healthy subjects (10/group) took part in the study. The two study groups were similar in terms of socio-demographic characteristics, age and gender (Table 1). Participants were randomly allocated to the 2 groups (ferrous sulphate or placebo) and asked to complete the gastrointestinal symptom questionnaire every day for 14 days. The completion rate of the symptom questionnaire was 100%. All participants found the symptom questionnaire easy to complete but some suggested that a ‘comments’ section be included so that they could, at times, provide reasons for responses. The average time for completion of the questionnaire was less than 5 minutes per day. Compliance with oral iron/placebo was >80% for the morning dose and >90% for the evening dose for participants in both groups and did not differ between groups. Reasons provided for non-compliance were unrelated to the study treatments.

**Table 1:** Baseline socio-demographic data for the participants in the 2 study groups

	**Iron group (n = 10)**	**Placebo group (n = 10)**
Age (years)		
Mean (SD)	32.2 (10.4)	31.7 (10.8)
Range	21-58	22-59
Gender [n]		
*Male*	3	4
*Female*	7	6
Highest education level [n (%)]		
*GCSE*	1 (10)	0
*A level*	2 (20)	4 (40)
*First degree or professional qualification*		
	4 (40)	3 (30)
*Higher degree*	3 (30)	3 (30)
Job title [n (%)]		
*Undergraduate student*	4 (40)	3 (30)
*PhD student*	2 (20)	1 (10)
*Secretary*	1 (10)	0
*Retired teacher*	0	1 (10)
*Dietician*	3 (30)	2 (20)
*Research fellow*	0	2 (20)
*Clinician*	0	1 (10)

Differences were not significant between the 2 groups, with *p* > 0.05.

**Figure 2:**
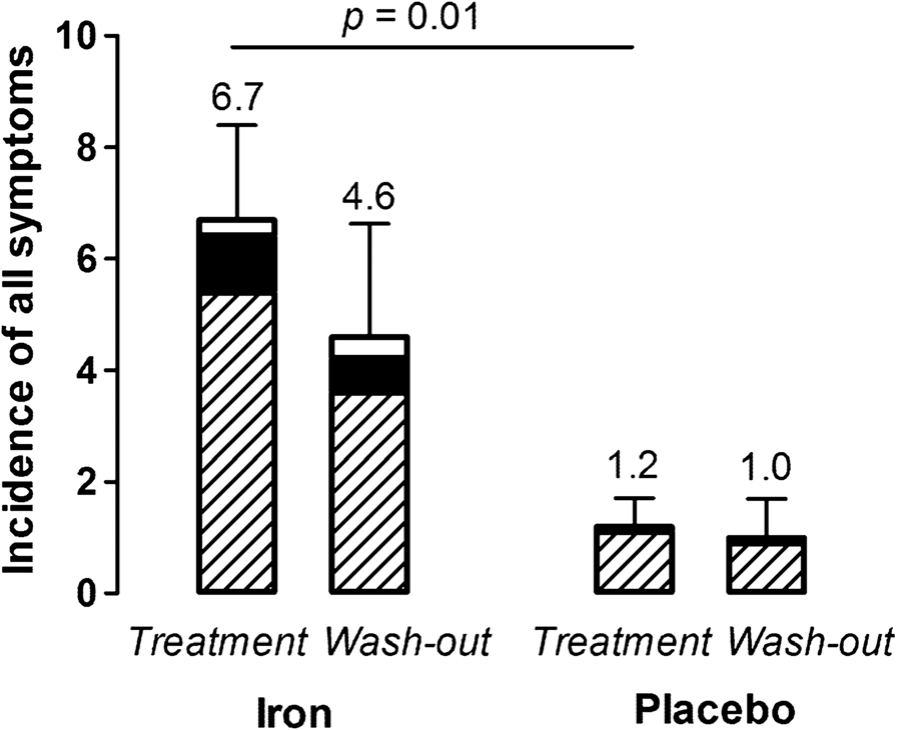
**Mean (SEM) incidence per participant of symptoms, from ‘box C’ of the questionnaire, in in week 1 (treatment) and week 2 (wash-out) of the iron and placebo groups.** The mean incidence of symptoms for the week as mild (stripped part of columns), moderate (closed) and severe (open) is shown.

In summary, a total of 12 (out of 20) participants reported the presence of one or more symptom(s) in the first week of the study (i.e. during treatment), of which 9 (75%) were in the ferrous sulphate group. In the second week of the study (i.e. wash-out), 9 participants reported one or more symptom(s), of which 6 (67%) were in the ferrous sulphate group. The degree of discomfort caused by each symptom was categorised into mild, moderate or severe as defined in box ‘C’ of the questionnaire (Figure 1). Detailed analyses were as follows: in the first week of the study (treatment) the number of symptoms reported per participant in the ferrous sulphate group (mean = 6.7, SEM = 1.7) was significantly higher than that reported per participant in the placebo group (mean = 1.2, SEM = 0.5) (*p* = 0.01). In the second week of the study (wash-out) the number of symptoms reported per participant in the ferrous sulphate group (mean = 4.6, SEM = 2.0) tended to be higher than that per participant in the placebo group (mean = 1.0, SEM = 0.7) but this did not reach significance (*p* = 0.12) (Figure 2).

**Figure 3:**
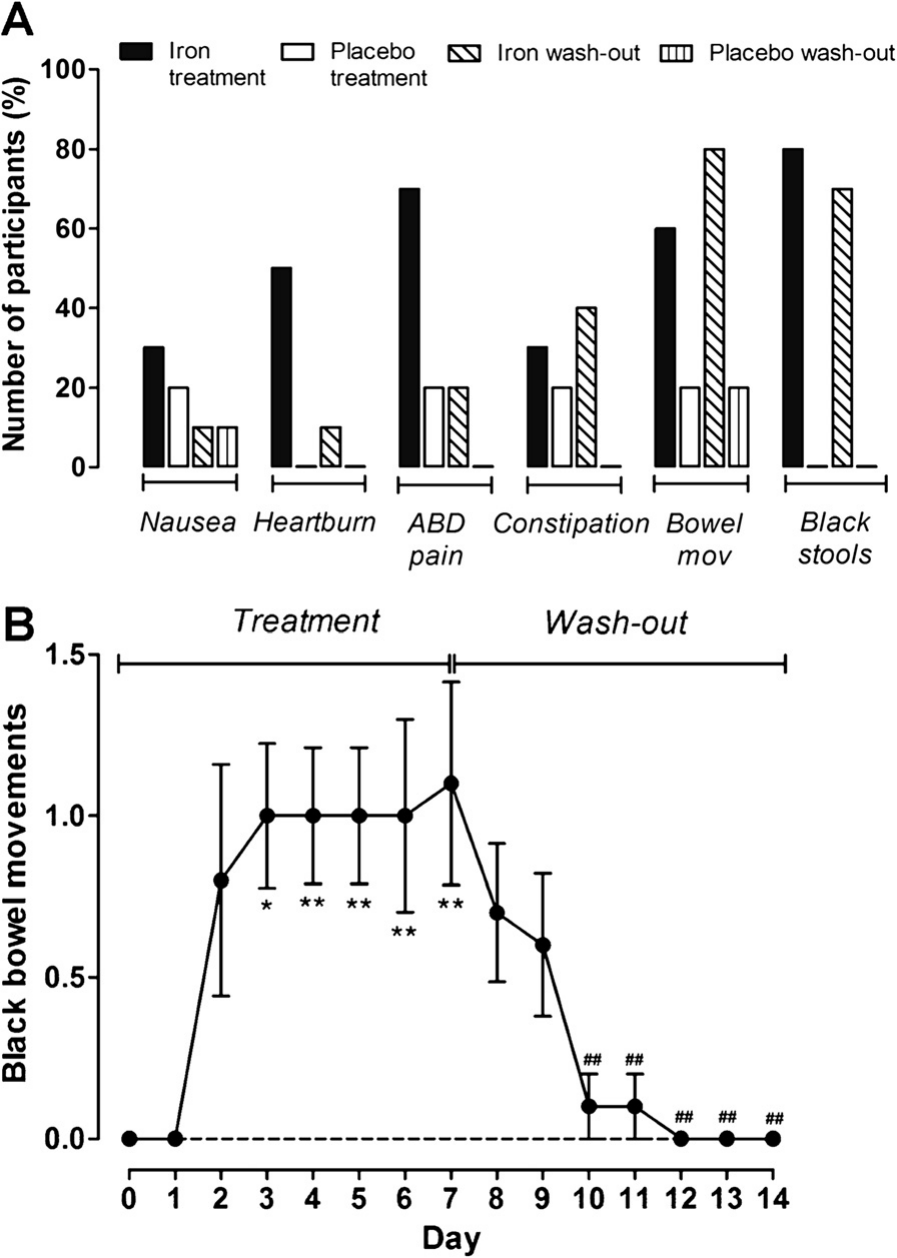
**Incidence of individual symptoms in the iron and placebo groups. A**, percentage of participants in each group reporting nausea, heartburn, abdominal pain (ABD pain), constipation, change in bowel movements (Bowel mov) or the presence of black stool movements on at least one day of each week of the intervention or wash-out periods (i.e. data from boxes ‘C’ and ‘H’ on the questionnaire (Figure 1)). The number of participants reporting symptoms in the iron group was significantly higher than that of the placebo group (*p* = 0.01). **B**, mean number of black bowel movements per day, obtained from box ‘H’ of the questionnaire (Figure 1), in the iron group (no black bowel movements were reported in the placebo group). Error bars represent standard error of mean (SEM). *p < 0.05; **p < 0.01 for comparison between each treatment day and the baseline (i.e. day 0). ^##^*p* < 0.01 for comparison between each wash-out day and the end of the treatment period (i.e. day 7).

Table 2 shows the reported frequency for individual symptoms by the participants in the ferrous sulphate and placebo groups during the treatment and wash-out periods. Adverse events for which the gastrointestinal symptom questionnaire was most discriminatory between ferrous sulphate and placebo groups were: heartburn, abdominal pain and the presence of black stools (all *p* ≤ 0.03) (Table 2). The differences observed in nausea, constipation and change in bowel movements between the ferrous sulphate and placebo groups may be clinically significant but, due to the small sample size, did not reach statistical significance (Table 2). The number of participants in each group reporting these individual symptoms is presented in Figure 3A, for both treatment and wash-out weeks. Vomiting was not reported by participants in either group on any day. Overall, the number of participants reporting symptoms in the iron group was significantly higher than the number of participants reporting symptoms in the placebo group (*p* = 0.01). The nature of the symptoms reported in the oral iron group is not surprising as iron that remains soluble in the intestinal lumen is likely to be bioavailable but, equally, may be available for other processes including uptake by commensal flora and colonic epithelial cells, and facile redox-cycling in the gut lumen. Indeed, the generation of harmful free-radicals through Fenton chemistry which can cause inflammation and oxidative stress in the intestinal mucosa has been linked to available luminal iron in several studies [53-55]. Furthermore, detrimental changes to the commensal microbiota have recently been reported with oral iron supplementation and may be partly responsible for the distal symptoms such as constipation and diarrhoea [27-29,56].

**Table 2:** Frequency of individual symptoms irrespective of whether mild, moderate or severe (days per week)

	**Iron**		**Placebo**		
**Symptom**	***Treatment***	***Wash-out***	***Treatment***	***Wash-out***	***p *** **value**^**#**^
**Nausea**	1.1 ± 0.6	0.1 ± 0.1	0.2 ± 0.1	0.4 ± 0.4	0.2
	(−0.3; 2.5)	(−0.1; 0.3)	(−0.1; 0.5)	(−0.5; 1.3)	
**Heartburn**	1.0 ± 0.4	0.3 ± 0.3	0	0	0.03
	(0.1; 1.9)	(−0.4; 1.0)	(0; 0)	(0; 0)	
**Abdominal pain**	2.0 ± 0.6	1.0 ± 0.7	0.2 ± 0.1	0	0.02
	(0.5; 3.5)	(−0.7; 2.7)	(−0.1; 0.5)	(0; 0)	
**Headache**	1.1 ± 0.5	1.6 ± 0.6	0.2 ± 0.2	0.1 ± 0.1	0.15
	(−0.1; 2.3)	(0.2; 3.0)	(−0.3; 0.7)	(−0.1; 0.3)	
**Breathlessness**	0.1 ± 0.1	0.5 ± 0.3	0	0.1 ± 0.1	0.3
	(−0.1; 0.3)	(−0.3; 1.3)	(0; 0)	(−0.1; 0.3)	
**Diarrohea**	0.4 ± 0.3	0.3 ± 0.2	0.3 ± 0.3	0.4 ± 0.2	0.8
	(−0.3; 1.1)	(−0.2; 0.8)	(−0.4; 1.0)	(−0.1; 0.9)	
**Constipation**	1.0 ± 0.6	0.7 ± 0.5	0.2 ± 0.2	0	0.2
	(−0.3; 2.3)	(−0.4; 1.8)	(−0.3; 0.7)	(0; 0)	
**Change bowel movements**	1.3 ± 0.5	1.5 ± 0.3	0.4 ± 0.3	0.4 ± 0.2	0.15
	(0.1; 2.5)	(0.7; 2.3)	(−0.2; 1.0)	(−0.1; 0.9)	
**Black stools**	4.1 ± 0.7	1.3 ± 0.4	0	0	0.0004
	(2.4; 5.8)	(0.4; 2.2)	(0; 0)	(0; 0)	
	*p* = 0.002				

Values, derived from boxes ‘C’ and ‘H’ of the questionnaire (Figure 1), are shown as mean ± standard error (95% confidence interval) for the two groups.

^#^significance value (*p* < 0.05) between the frequency of each symptom for the Iron treatment period versus the Placebo treatment period.

Figure 3B shows the number of black stool movements reported in the ferrous sulphate group: no black stools were reported by participants in the placebo group. For those participants taking ferrous sulphate there was a significant increase in the number of black stool movements from day 3 of the treatment period (*p* = 0.03) which only returned to baseline 3 days after stopping the ferrous sulphate tablets (i.e. day 10 of the study) (*p* = 0.005) (Figure 3B). Although subjects were not aware of which group they were in (i.e. treatment or placebo), the presence of black stools could have provided an indication of their randomisation to oral iron. It is thus important to note that symptoms were being reported in the ferrous sulphate group from the first day (for example nausea and abdominal pain) and this was before the onset of black stools which was significant at 72 hours.

The blackening of stools following oral iron supplementation is derived from the reaction of hydrogen sulphide produced by commensal bacteria, such as sulphate-reducing bacteria, with ferrous iron (Fe^2+^) to produce iron sulphide (FeS). Sulphate-reducing bacteria are a group of ‘emerging pathogens’ in the colon. These bacteria have an exceptionally high requirement for iron and use sulphur or sulphate, which are plentiful in the colon from the digestion of foods, as electron acceptors during anaerobic respiration, for the oxidative metabolism of organic compounds and H_2_[57]. Indeed, Werner et al. have shown that numbers of *Desulfovibrio* (the main sulphate-reducing bacteria found in faeces) significantly increased following ferrous sulphate supplementation [28].

## Conclusions

A short and simple-to-answer gastrointestinal symptom questionnaire was developed as a tool to measure adverse-effects for use in intervention studies with oral iron and especially when ferrous sulphate comprises one study arm. The present small study aimed to show where the questionnaire had discriminatory power: i.e. which symptoms are more sensitive to oral ferrous sulphate therapy. The questionnaire was filled in by the study participants during the 7-days of the treatment period and the 7-days of the wash-out period and was discriminant in the total number of reported symptoms per participant between the iron and the placebo groups. Additionally, it showed that the individual symptoms that were significantly more sensitive to the oral iron treatment were heartburn, abdominal pain and the presence of black stools. Although there has been concern that black stools caused by oral iron may mask black stools caused by gastrointestinal blood loss, it is not per se an adverse-effect related to symptoms but, rather, it represents the formation of iron sulphide in the colon due to bacterial activity. However, it could be useful as a marker of the change in bacterial flora (i.e. an increase in sulphate-reducing bacteria at the expense of beneficial bacterial genus such as lactobacilli) in future intervention studies with oral iron and this could be further investigated.

Despite the uncomplicated format of the questionnaire, a large amount of data is generated. For example, in this small study, 20 participants provided 14 days of questionnaire each (i.e. 280 questionnaires) with 28 pieces of information per questionnaire: this represents 7,840 pieces of data recorded, for most, in a categorical format, for some in a binary form, and for others in a continuous format.

To facilitate analysis, the main outcomes were restricted to binary events (e.g. presence and absence of symptoms), and the categorisation of their severity was only focussed on data collected from boxes C, H and I of the questionnaire. Clearly, in future studies that utilise this questionnaire, researchers will need to determine whether (a) they collect all data or simplify the questionnaire even further so that only C, H and I are used and (b) if they collect all, how it should be best analysed.Finally, whether the whole questionnaire (Figure 1) is used, or a short version with only boxes C, H and I, a comments box may be a useful addition for patients/participants to provide detail that may inform upon their responses.

## Competing interests

The authors declare no conflict of interest but DIAP and JJP wish to note that they are co-inventors on a patent detailing novel Fe(III) poly oxo-hydroxide structures that may have potential as dietary supplements and that they consult to Iron Therapeutics UK Limited for advice on their novel oral iron therapeutic.

## Authors’ contributions

MCEL and JJP designed the research; MCEL conducted the research; DIAP and SMI analysed data; DIAP, SMI and JJP wrote the paper; DIAP and JJP had primary responsibility for final content. All authors read and approved the final manuscript.

## Pre-publication history

The pre-publication history for this paper can be accessed here:

http://www.biomedcentral.com/1471-230X/14/103/prepub

## References

[B1] WHO The Global Burden of Disease: 2004 Update 2008 Geneva: WHO 1 146

[B2] WHO The World Health Report. Reducing Risks, Promoting Healthy Life 2002 Geneva: WHO

[B3] Ezzati M Lopez AD Rodgers A Vander Hoorn S Murray CJ Selected major risk factors and global and regional burden of disease Lancet 2002 360 1347 1360 12423980 10.1016/S0140-6736(02)11403-6

[B4] Joint Formulary Committee British National Formulary 2014 London: British Medical Association: Pharmaceutical Society of Great Britain

[B5] de Souza AI Batista Filho M Bresani CC Ferreira LO Figueiroa JN Adherence and side effects of three ferrous sulfate treatment regimens on anemic pregnant women in clinical trials Cad Saude Publica 2009 25 1225 1233 19503953 10.1590/s0102-311x2009000600005

[B6] Milman N Byg KE Bergholt T Eriksen L Side effects of oral iron prophylaxis in pregnancy–myth or reality? Acta Haematol 2006 115 53 57 16424650 10.1159/000089466

[B7] Galloway R McGuire J Determinants of compliance with iron supplementation: supplies, side effects, or psychology? Soc Sci Med 1994 39 381 390 7939855 10.1016/0277-9536(94)90135-x

[B8] de Silva AD Tsironi E Feakins RM Rampton DS Efficacy and tolerability of oral iron therapy in inflammatory bowel disease: a prospective, comparative trial Aliment Pharmacol Ther 2005 22 1097 1105 16305723 10.1111/j.1365-2036.2005.02700.x

[B9] Idoate Gastearena MA Gil AG Azqueta A Coronel MP Gimeno M A comparative study on the gastroduodenal tolerance of different antianaemic preparations Hum Exp Toxicol 2003 22 137 141 12723894 10.1191/0960327103ht330oa

[B10] Mohamed HJ Vidas N Mudway IS Liu DY Geissler CA Powell JJ Lomer MC Oral ferrous sulphate tolerance study in healthy individuals (Poster) TEMA 12 Symposium 2005 Northern Ireland, UK: University of Ulster at Coleraine

[B11] Sazawal S Black RE Ramsan M Chwaya HM Stoltzfus RJ Dutta A Dhingra U Kabole I Deb S Othman MK Kabole FM Effects of routine prophylactic supplementation with iron and folic acid on admission to hospital and mortality in preschool children in a high malaria transmission setting: community-based, randomised, placebo-controlled trial Lancet 2006 367 133 143 16413877 10.1016/S0140-6736(06)67962-2

[B12] Drakesmith H Prentice A Viral infection and iron metabolism Nat Rev Microbiol 2008 6 541 552 18552864 10.1038/nrmicro1930

[B13] Friedman JF Kurtis JD Kabyemela ER Fried M Duffy PE The iron trap: iron, malaria and anemia at the mother-child interface Microbes Infect 2009 11 460 466 19285567 10.1016/j.micinf.2009.02.006

[B14] WHO Conclusions and recommendations of the WHO Consultation on prevention and control of iron deficiency in infants and young children in malaria-endemic areas Food Nutr Bull 2007 28 S621 S627 18297899 10.1177/15648265070284s414

[B15] GP prescribing data [http://www.hscic.gov.uk/gpprescribingdata]

[B16] Ruivard M Feillet-Coudray C Rambeau M Gerbaud L Mazur A Rayssiguier Y Philippe P Coudray C Effect of daily versus twice weekly long-term iron supplementation on iron absorption and status in iron-deficient women: a stable isotope study Clin Biochem 2006 39 700 707 16603147 10.1016/j.clinbiochem.2006.02.008

[B17] Siddiqui IA Rahman MA Jaleel A Efficacy of daily vs. weekly supplementation of iron in schoolchildren with low iron status J Trop Pediatr 2004 50 276 278 15510758 10.1093/tropej/50.5.276

[B18] Fischer Walker CL Baqui AH Ahmed S Zaman K El Arifeen S Begum N Yunus M Black RE Caulfield LE Low-dose weekly supplementation of iron and/or zinc does not affect growth among Bangladeshi infants Eur J Clin Nutr 2009 63 87 92 17882136 10.1038/sj.ejcn.1602905

[B19] Hyder SM Persson LA Chowdhury AM Ekstrom EC Do side-effects reduce compliance to iron supplementation? A study of daily- and weekly-dose regimens in pregnancy J Health Popul Nutr 2002 20 175 179 12186198

[B20] Stoltzfus RJ Chway HM Montresor A Tielsch JM Jape JK Albonico M Savioli L Low dose daily iron supplementation improves iron status and appetite but not anemia, whereas quarterly anthelminthic treatment improves growth, appetite and anemia in Zanzibari preschool children J Nutr 2004 134 348 356 14747671 10.1093/jn/134.2.348

[B21] Makrides M Crowther CA Gibson RA Gibson RS Skeaff CM Efficacy and tolerability of low-dose iron supplements during pregnancy: a randomized controlled trial Am J Clin Nutr 2003 78 145 153 12816784 10.1093/ajcn/78.1.145

[B22] Shatrugna V Raman L Kailash U Balakrishna N Rao KV Effect of dose and formulation on iron tolerance in pregnancy Natl Med J India 1999 12 18 20 10326325

[B23] Little BB Pharmacokinetics during pregnancy: evidence-based maternal dose formulation Obstet Gynecol 1999 93 858 868 10912434 10.1016/s0029-7844(98)00444-x

[B24] Hutchinson C Al-Ashgar W Liu DY Hider RC Powell JJ Geissler CA Oral ferrous sulphate leads to a marked increase in pro-oxidant nontransferrin-bound iron Eur J Clin Invest 2004 34 782 784 15530152 10.1111/j.1365-2362.2004.01416.x

[B25] Erichsen K Ulvik RJ Grimstad T Berstad A Berge RK Hausken T Effects of ferrous sulphate and non-ionic iron-polymaltose complex on markers of oxidative tissue damage in patients with inflammatory bowel disease Aliment Pharmacol Ther 2005 22 831 838 16225492 10.1111/j.1365-2036.2005.02652.x

[B26] Evans P Halliwell B Micronutrients: oxidant/antioxidant status Br J Nutr 2001 85 Suppl 2 S67 S74 11509092

[B27] Dostal A Chassard C Hilty FM Zimmermann MB Jaeggi T Rossi S Lacroix C Iron depletion and repletion with ferrous sulfate or electrolytic iron modifies the composition and metabolic activity of the gut microbiota in rats J Nutr 2012 142 271 277 22190022 10.3945/jn.111.148643 PMC3260059

[B28] Werner T Wagner SJ Martinez I Walter J Chang JS Clavel T Kisling S Schuemann K Haller D Depletion of luminal iron alters the gut microbiota and prevents Crohn’s disease-like ileitis Gut 2011 60 325 333 21076126 10.1136/gut.2010.216929

[B29] Zimmermann MB Chassard C Rohner F N’Goran EK Nindjin C Dostal A Utzinger J Ghattas H Lacroix C Hurrell RF The effects of iron fortification on the gut microbiota in African children: a randomized controlled trial in Cote d’Ivoire Am J Clin Nutr 2010 92 1406 1415 20962160 10.3945/ajcn.110.004564

[B30] Crane SJ Talley NJ Chronic gastrointestinal symptoms in the elderly Clin Geriatr Med 2007 23 721 734 v 17923334 10.1016/j.cger.2007.06.003

[B31] Kearney DJ McDermott K Martinez M Simpson TL Association of participation in a mindfulness programme with bowel symptoms, gastrointestinal symptom-specific anxiety and quality of life Aliment Pharmacol Ther 2011 34 363 373 21651595 10.1111/j.1365-2036.2011.04731.x

[B32] Jerndal P Ringstrom G Agerforz P Karpefors M Akkermans LM Bayati A Simren M Gastrointestinal-specific anxiety: an important factor for severity of GI symptoms and quality of life in IBS Neurogastroenterol Motil 2010 22 646 e179 20367800 10.1111/j.1365-2982.2010.01493.x

[B33] Portincasa P Maggipinto A Berardino M Bonfrate L Costin S Todarello O Palasciano G Wang DQ Dumitrascu DL Assessing gastrointestinal symptoms and perception, quality of life, motility, and autonomic neuropathy in clinical studies J Gastrointestin Liver Dis 2009 18 205 211 19565052

[B34] Jeong JJ Choi MG Cho YS Lee SG Oh JH Park JM Cho YK Lee IS Kim SW Han SW Choi KY Chung IS Chronic gastrointestinal symptoms and quality of life in the Korean population World J Gastroenterol 2008 14 6388 6394 19009657 10.3748/wjg.14.6388 PMC2766123

[B35] Brunner HI Johnson AL Barron AC Passo MH Griffin TA Graham TB Lovell DJ Gastrointestinal symptoms and their association with health-related quality of life of children with juvenile rheumatoid arthritis: validation of a gastrointestinal symptom questionnaire J Clin Rheumatol 2005 11 194 204 16357756 10.1097/01.rhu.0000173616.81928.44

[B36] Bovenschen HJ Laheij RJ Tan AC Witteman EM Rossum LG Jansen JB Health-related quality of life of patients with gastrointestinal symptoms Aliment Pharmacol Ther 2004 20 311 319 15274668 10.1111/j.1365-2036.2004.02076.x

[B37] Wolfe F Kong SX Watson DJ Gastrointestinal symptoms and health related quality of life in patients with arthritis J Rheumatol 2000 27 1373 1378 10852256

[B38] Schultink W van der Ree M Matulessi P Gross R Low compliance with an iron-supplementation program: a study among pregnant women in Jakarta, Indonesia Am J Clin Nutr 1993 57 135 139 8424380 10.1093/ajcn/57.2.135

[B39] Lutsey PL Dawe D Villate E Valencia S Lopez O Iron supplementation compliance among pregnant women in Bicol, Philippines Public Health Nutr 2008 11 76 82 17519048 10.1017/S1368980007000237

[B40] Seck BC Jackson RT Determinants of compliance with iron supplementation among pregnant women in Senegal Public Health Nutr 2008 11 596 605 17764606 10.1017/S1368980007000924

[B41] Armstrong D Veldhuyzen SJ Chung SA Shapiro CM Dhillon S Escobedo S Chakraborty BK Mann V Tanser L Nevin K Validation of a short questionnaire in English and French for use in patients with persistent upper gastrointestinal symptoms despite proton pump inhibitor therapy: the PASS (Proton pump inhibitor Acid Suppression Symptom) test Can J Gastroenterol 2005 19 350 358 15997268 10.1155/2005/569368

[B42] Dimenas E Carlsson G Glise H Israelsson B Wiklund I Relevance of norm values as part of the documentation of quality of life instruments for use in upper gastrointestinal disease Scand J Gastroenterol 1996 31 8 13 10.3109/00365529609095544 9110389

[B43] Dimenas E Glise H Hallerback B Hernqvist H Svedlund J Wiklund I Well-being and gastrointestinal symptoms among patients referred to endoscopy owing to suspected duodenal-ulcer Scand J Gastroenterol 1995 30 1046 1052 8578162 10.3109/00365529509101605

[B44] Talley NJ Boyce PM Owen BK Newman P Paterson KJ Initial validation of a bowel symptom questionnaire and measurement of chronic gastrointestinal symptoms in Australians Aust N Z J Med 1995 25 302 308 8540870 10.1111/j.1445-5994.1995.tb01894.x

[B45] Foster JM van der Molen T Caeser M Hannaford P The use of questionnaires for measuring patient-reported side effects of drugs: its importance and methodological challenges Pharmacoepidemiol Drug Saf 2008 17 278 296 18300325 10.1002/pds.1533

[B46] Varma MG Wang JY Berian JR Patterson TR McCrea GL Hart SL The constipation severity instrument: a validated measure Dis Colon Rectum 2008 51 162 172 18172725 10.1007/s10350-007-9140-0

[B47] Quan C Talley NJ Cross S Jones M Hammer J Giles N Horowitz M Development and validation of the diabetes bowel symptom questionnaire Aliment Pharmacol Ther 2003 17 1179 1187 12752355 10.1046/j.1365-2036.2003.01553.x

[B48] Nappi CTG Morra I Massaro M Formisano C Di Carlo C Efficacy and tolerability of oral bovine lactoferrin compared to ferrous sulfate in pregnant women with iron deficiency anemia: a prospective controlled randomized study Acta Obstet Gynecol Scand 2009 88 1031 1035 19639462 10.1080/00016340903117994

[B49] Sifakis S Angelakis E Papadopoulou E Stratoudakis G Fragouli Y Koumantakis E The efficacy and tolerability of iron protein succinylate in the treatment of iron-deficiency anemia in pregnancy Clin Exp Obstet Gynecol 2005 32 117 122 16108396

[B50] Aamodt AH Stovner LJ Hagen K Zwart JA Comorbidity of headache and gastrointestinal complaints. The Head-HUNT Study Cephalalgia 2008 28 144 151 18197884 10.1111/j.1468-2982.2007.01486.x

[B51] Spierings EL Headache of gastrointestinal origin: case studies Headache 2002 42 217 219 11903545 10.1046/j.1526-4610.2002.02054.x

[B52] Pedersen A Sandstrom B Van Amelsvoort JM The effect of ingestion of inulin on blood lipids and gastrointestinal symptoms in healthy females Br J Nutr 1997 78 215 222 9301412 10.1079/bjn19970141

[B53] Carrier J Aghdassi E Cullen J Allard JP Iron supplementation increases disease activity and vitamin E ameliorates the effect in rats with dextran sulfate sodium-induced colitis J Nutr 2002 132 3146 3150 12368409 10.1093/jn/131.10.3146

[B54] Lund EK Wharf SG Fairweather-Tait SJ Johnson IT Oral ferrous sulfate supplements increase the free radical-generating capacity of feces from healthy volunteers Am J Clin Nutr 1999 69 250 255 9989688 10.1093/ajcn/69.2.250

[B55] Orozco MN Solomons NW Schumann K Friel JK de Montenegro AL Antioxidant-rich oral supplements attenuate the effects of oral iron on in situ oxidation susceptibility of human feces J Nutr 2010 140 1105 1110 20392879 10.3945/jn.109.111104

[B56] Kortman GA Boleij A Swinkels DW Tjalsma H Iron availability increases the pathogenic potential of Salmonella typhimurium and other enteric pathogens at the intestinal epithelial interface PLoS One 2012 7 e29968 22272265 10.1371/journal.pone.0029968 PMC3260200

[B57] Gibson GR Physiology and ecology of the sulphate-reducing bacteria J Appl Bacteriol 1990 69 769 797 2286579 10.1111/j.1365-2672.1990.tb01575.x

